# Race and insurance status outcome disparities following splenectomy in trauma patients are reduced in larger hospitals. A cross-sectional study

**DOI:** 10.1016/j.amsu.2022.103516

**Published:** 2022-04-08

**Authors:** Harrison J. Kaplan, I. Michael Leitman

**Affiliations:** Department of Surgery, Icahn School of Medicine at Mount Sinai, One Gustave L. Levy Place, Box 1076, New York, NY, 10029, USA

**Keywords:** Spleen, Splenectomy, Trauma, Race, Insurance

## Abstract

**Background:**

Splenectomy, still a commonly performed treatment for splenic injury in trauma patients, has been shown to have a high rate of complications. The purpose of this study was to identify predictors, including race and insurance status, associated with adverse outcomes post-splenectomy in trauma patients. We discuss possible explanations and methods for reducing these disparities.

**Methods:**

The American College of Surgeons – Trauma Quality Improvement Program (ACS-TQIP) participant user database was queried from 2010 to 2015 and patients who underwent total splenectomy were identified. All mechanisms of injury, including both blunt and penetrating trauma, were included. Patients with advance directives limiting care or aged under 18 were excluded. Propensity score matching was used to control for age, preexisting medical conditions, and the severity of the traumatic injury. A chi-squared test was used to find significant associations between available predictors and outcomes for this cross-sectional study.

**Results:**

The post-splenectomy mortality rate was 9.2% (n = 1047), 8.0% (n = 918) of patients had three or more complications, and 20.3% (n = 2315) had major complications. A primary race of white (OR 0.7, 95% Confidence Interval (CI) 0.6–0.9, p < 0.01) and private insurance (OR 0.5, 95%CI 0.4–0.6, p < 0.01) were associated with lower risks of mortality A primary race of neither Black nor white (OR 1.3, 95%CI 1.03–1.7, p = 0.03) and a lack of health insurance (“self-pay”) (OR 1.6, 95%CI 1.3–1.9, p < 0.01) were both correlated with mortality. When limited to hospitals of 600+ beds, there were no associations between race and mortality.

**Conclusion:**

The post-splenectomy mortality rate after trauma remains high. In U.S. trauma centers, a primary race of Black and payment status of “self-pay” are associated with adverse outcomes after splenectomy following a traumatic injury. These disparities are reduced when limiting analysis to larger hospitals. Efforts to reduce disparities in outcomes among trauma patients requiring a splenectomy should focus on improving resource availability and quality in smaller hospitals.

## Highlights

• The post-splenectomy mortality rate in trauma patients remains high.
• Mortality is less frequent in white patients, and more frequent in uninsured patients.
• Black patients were more likely to experience major complications following splenectomy.
• In hospitals with greater than 600 beds, there were no associations between race and mortality following splenectomy.

## 1 Introduction

Total splenectomy is still commonly performed as the treatment for splenic injury, even as nonoperative management (NOM) has become the first choice for hemodynamically stable patients at many trauma centers. NOM greatly reduces rates of nontherapeutic laparotomies and thus rates of complications, length of stay, and overall cost [1]. NOM generally consists of close observation of patients, with packed red blood cell transfusion and angioembolization to control bleeding if necessary [2].This consensus around the benefits of NOM was first reached for the management of blunt splenic trauma, although it has been shown that this paradigm is also useful for managing patients with splenic injuries from penetrating trauma [3]. Navsaria et al. [3] successfully used NOM in patients with abdominal gunshot wounds at Groote Schuur Hospital in Cape Town, South Africa, with less than 5% requiring delayed surgical management [3]. The rate of unnecessary laparotomies in trauma centers when performed on all patients with penetrating trauma is as high as 37% [4]. While NOM can be used to successfully lower rates of unnecessary laparotomies in trauma patients, there will ultimately still be patients who require a laparotomy and splenectomy for successful treatment of their injuries with the technology that is currently available.

Post-splenectomy patients are at high risk for complications, especially infection and sepsis in the long term [5]. The spleen plays an important role in the immune system, and it is well known that patients with asplenia have a much greater risk of infection, especially from encapsulated bacteria. This is true for post-splenectomy patients or those suffering from conditions that can cause auto-splenectomy, such as sickle cell anemia [6]. Splenectomy itself has also been shown to have a high risk of short-term complications [7]. Previous studies have found that certain underlying conditions, such as myelofibrosis, and other factors, such as age, are associated with an increased risk of complications following splenectomy, though this was not always in the context of trauma [[8], [9], [10]]. The purpose of this study is to identify additional predictors, including racial and socioeconomic factors, associated with an increased mortality rate and other adverse outcomes in patients who are undergoing splenectomy for traumatic injury. Further, it has been shown that controlling for other factors associated with hospital quality can reduce racial disparities in outcomes, and we hypothesize that the same holds true for trauma patients post-splenectomy [11]. Race itself is not a risk factor or a biological determinant but is rather associated with a multitude of other factors that affect medical outcomes [12].

## 2 Methods

This study was reviewed by the Institutional Review Board of the Icahn School of Medicine at Mount Sinai in New York, NY (IRB-20-03069), including a waiver of patient consent. Retrospective cohort study used the American College of Surgeons (ACS) Trauma Quality Improvement Program (TQIP) participant user database from 2010 to 2015. In accordance with the Declaration of Helsinki, the research was registered with ResearchRegistry.com (https://www.researchregistry.com, Research Registry Unique Identifying Number 7562).

We performed a query for patients who underwent a total splenectomy at a participating verified trauma center. Patients who underwent a total splenectomy were identified by checking all available procedure codes for International Classification of Disease (ICD-9), code 41.5. Patients under the age of 18 or those who had advance directives were excluded. Patients who underwent a partial splenectomy or splenorraphy were not included. Both blunt and penetrating mechanisms of injury were included, and no exclusions were made based on trauma center level.

Available demographic data in the ACS-TQIP database included gender, race, ethnicity, payment status, and region. Race was grouped into Black, white, or other, which was consistent with previously published studies using the TQIP dataset [13]. The geographical region refers to the location within the United States of the trauma center where the patient received treatment. All available comorbidities were collected, as well as clinical data points including Injury Severity Score (ISS) and initial systolic blood pressure (SBP). ISS was calculated using the available Abbreviated Injury Scale (AIS) scores.

### 2.1 Outcomes

The primary objective was to determine if any of the available demographic factors were associated with our primary or secondary end points after a splenectomy in trauma patients. The primary end point was mortality, and the secondary end points were three or more complications or any major complication. Complications that would likely be classified as grade IV or greater in the classification system proposed by Dindo et al. [14]. were considered to be major complications and included: acute kidney injury, acute respiratory distress syndrome, cardiac arrest with Cardiopulmonary Resuscitation (CPR), myocardial infarction, pulmonary embolism, stroke/cerebrovascular accident (CVA), unplanned intubation, unplanned return to ICU, and severe sepsis. This cross-sectional study only considered end points that occurred prior to initial discharge.

### 2.2 Data Analysis

A total of 11,753 patients were identified who underwent a total splenectomy at a participating trauma center between January 2010 and December 2015. Of these, 334 records were missing necessary clinical data or demographics, leaving 11,419 valid patient records for review and analysis. Propensity score matching was used to control for age, preexisting medical conditions, and the severity of the traumatic injury. Previous work has shown that a strong propensity score model can be created by selecting variables that are likely related to the outcome but not the risk factor being studied [15]. We chose to use all available comorbidities, as well as Injury Severity Score (ISS) and initial SBP in our propensity score model. The final propensity score model included: age, ISS, initial SBP, alcohol use disorder, ascites in the prior 30 days, bleeding disorder, chemotherapy, congenital defects, congestive heart failure (CHF), current smoker, dialysis, cerebrovascular accident with deficit, diabetic, disseminated cancer, esophageal varices, functionally dependent, angina in the prior six months, peripheral vascular disease, hypertension, impaired sensorium, premature birth, obesity, respiratory disease, steroid use, cirrhosis, dementia, major psychiatric disorder, prehospital arrest with CPR, and attention deficit disorder.

Matching was then performed based on propensity scores using a greedy algorithm with a 1:2 ratio. For example, one patient who died was matched with two controls. The above propensity score matching process was repeated for each of the secondary end points.

Previous studies using similar propensity score matching methods have used the standardized mean difference to assess for balance after matching, using a maximum of 0.1 as a strong threshold [16]. The standardized mean differences for all variables used in our propensity score matching model were below this threshold, suggesting that groups were balanced. A comparison of demographics and other factors between the two groups in each of the matched cohorts was performed using a chi-squared test. We then repeated the identical propensity matching and analysis process while limiting our dataset to the 5931 patients treated at high-volume hospitals, which we defined as hospitals with more than 600 adult beds.

All statistical methods were performed on Statistical Analysis Software version 9.4 (SASv9.4, SAS Institute, Cary, NC). A value of p < 0.05 was considered to be significant. This work has been reported in line with the STROCSS criteria [17].

## 3 Results

The post-splenectomy mortality rate was 9.2% (n = 1047), 8.0% (n = 918) had three or more complications, and 20.3% (n = 2315) had major complications. After propensity score matching, 1042 patients who died prior to discharge were matched with 2072 controls, 914 patients who experienced three or more complications were matched with 1805 controls, and 2172 patients who experienced major complications were matched with 4336 controls. A summary of characteristics before and after matching can be found in Table 1, Table 2.

**Table 1:** Baseline characteristics before and after matching expired patients following splenectomy.

Variables	Pre-Matching					Post-Matching				
	Expired (*n* = 1047)		Controls (*n* = 10,372)		SMD	Expired (*n* = 1042)		Controls (n = 2070)		SMD
Age, y	48.4	[20.89]	41.2	[17.20]	0.380	48.4	(20.9)	48.9	(19.1)	0.024
Gender, male	718	(68.6)	7425	(71.6)	0.066	715	(68.6)	1432	(69.0)	0.009
Injury Severity Score	37.5	[18.72]	30.3	[18.42]	0.384	37.4	(18.7)	37.4	(17.6)	0.002
Systolic Blood Pressure	99.8	[42.60]	114.1	[30.31]	0.386	99.9	(42.7)	100.8	(32.2)	0.024
Attention Deficit Disorder	0	(0.0)	977	(9.4)	0.077	0	(0.0)	0	(0.0)	
Alcohol Use Disorder	56	(5.3)	23	(0.2)	0.156	55	(5.3)	115	(5.5)	0.012
Angina	0	(0.0)	376	(3.6)	0.044	0	(0.0)	0	(0.0)	
Ascites	8	(0.8)	18	(0.2)	0.077	6	(0.6)	14	(0.7)	0.013
Bleeding Disorder	59	(5.6)	21	(0.2)	0.096	59	(5.7)	120	(5.8)	0.005
Chemotherapy	3	(0.3)	131	(1.3)	0.024	3	(0.3)	4	(0.2)	0.019
Congestive Heart Failure	30	(2.9)	2405	(23.2)	0.113	29	(2.8)	60	(2.9)	0.007
Cirrhosis	37	(3.5)	60	(0.6)	0.146	34	(3.3)	62	(3.0)	0.016
Congenital Defect	3	(0.3)	86	(0.8)	0.017	3	(0.3)	4	(0.2)	0.019
Smoker	70	(6.7)	663	(6.4)	0.476	70	(6.7)	147	(7.1)	0.015
CVA/Stroke	20	(1.9)	41	(0.4)	0.093	20	(1.9)	43	(2.1)	0.011
Dementia	5	(0.5)	20	(0.2)	0.022	5	(0.5)	10	(0.5)	0.000
Functionally Dependent	5	(0.5)	53	(0.5)	0.005	5	(0.5)	6	(0.3)	0.031
Diabetic	80	(7.6)	10	(0.1)	0.049	79	(7.6)	154	(7.4)	0.006
Dialysis	8	(0.8)	119	(1.1)	0.023	8	(0.8)	14	(0.7)	0.011
Disseminated Cancer	11	(1.1)	24	(0.2)	0.077	11	(1.1)	18	(0.9)	0.019
Drug Use	18	(1.7)	1822	(17.6)	0.341	18	(1.7)	36	(1.7)	0.001
Esophageal Varices	5	(0.5)	3	(0.0)	0.049	4	(0.4)	11	(0.5)	0.022
Hypertension	184	(17.6)	642	(6.2)	0.000	184	(17.6)	384	(18.5)	0.022
Major Psychiatric Disorder	22	(2.1)	570	(5.5)	0.219	22	(2.1)	56	(2.7)	0.038
Myocardial Infarction	24	(2.3)	29	(0.3)	0.088	24	(2.3)	61	(2.9)	0.040
Obesity	53	(5.1)	134	(1.3)	0.049	53	(5.1)	108	(5.2)	0.005
Pre-Hospital Arrest	16	(1.5)	35	(0.3)	0.138	15	(1.4)	20	(1.0)	0.044
Premature	0	(0.0)	678	(6.5)	0.024	0	(0.0)	0	(0.0)	
Peripheral Vascular Disease	2	(0.2)	978	(9.4)	0.009	2	(0.2)	2	(0.1)	0.025
Respiratory Disease	53	(5.1)	25	(0.2)	0.019	53	(5.1)	108	(5.2)	0.005
Steroid Use	4	(0.4)	31	(0.3)	0.018	4	(0.4)	4	(0.2)	0.036

Data are presented as number (percentage) or mean [standard deviation].

SMD, standardized mean difference, CVA, Cerebrovascular Accident.

**Table 2:** Baseline characteristics before and after matching patients with major complications following splenectomy.

Variables	Pre-Matching					Post-Matching				
	Expired (*n* = 2315)		Controls (*n* = 9104)		SMD	Expired (*n* = 2172)		Controls (*n* = 4336)		SMD
Age, y	46.4	[18.75]	40.7	[17.22]	0.3195	45.3	[18.53]	45.1	[18.22]	0.0139
Gender, male	1703	(73.6)	6440	(70.7)	0.0631	1597	(73.5)	3181	(73.4)	0.0037
Injury Severity Score	35.1	[17.78]	29.9	[18.61]	0.2827	35.5	[17.64]	35.7	[17.49]	0.0091
Systolic Blood Pressure	109.4	[36.20]	113.7	[30.66]	0.1261	109.3	[36.51]	109.3	[31.83]	0.0017
ADD	4	(0.0)	27	(0.3)	0.0256	4	(0.2)	7	(0.2)	0.0055
Alcohol Use Disorder	252	(10.9)	781	(8.6)	0.0779	243	(11.2)	458	(10.6)	0.0201
Angina	4	(0.2)	6	(0.1)	0.0310	2	(0.1)	6	(0.1)	0.0136
Ascites	16	(0.7)	15	(0.2)	0.0807	15	(0.7)	15	(0.3)	0.0480
Bleeding Disorder	154	(6.7)	281	(3.1)	0.1662	123	(5.7)	218	(5.0)	0.0282
Chemotherapy	5	(0.2)	16	(0.2)	0.0091	4	(0.2)	7	(0.2)	0.0055
CHF	67	(2.9)	94	(1.0)	0.1345	59	(2.7)	82	(1.9)	0.0550
Cirrhosis	63	(2.7)	108	(1.2)	0.1111	58	(2.7)	94	(2.2)	0.0327
Congenital Defect	10	(0.4)	14	(0.2)	0.0515	8	(0.4)	12	(0.3)	0.0161
Smoker	438	(18.9)	2037	(22.4)	0.0854	409	(18.8)	806	(18.6)	0.0062
CVA/Stroke	41	(1.8)	65	(0.7)	0.0955	29	(1.3)	55	(1.3)	0.0059
Dementia	14	(0.6)	26	(0.3)	0.0479	13	(0.6)	22	(0.5)	0.0123
Functionally Dependent	19	(0.8)	39	(0.4)	0.0498	17	(0.8)	32	(0.7)	0.0051
Diabetic	242	(10.5)	501	(5.5)	0.1834	214	(9.9)	390	(9.0)	0.0294
Dialysis	20	(0.9)	48	(0.5)	0.0405	19	(0.9)	37	(0.9)	0.0023
Disseminated Cancer	21	(0.9)	31	(0.3)	0.0720	17	(0.8)	25	(0.6)	0.0251
Drug Use	148	(6.4)	848	(9.3)	0.1087	144	(6.6)	270	(6.2)	0.0164
Esophageal Varices	11	(0.5)	14	(0.2)	0.0574	10	(0.5)	13	(0.3)	0.0261
Hypertension	579	(25.0)	1427	(15.7)	0.2335	495	(22.8)	945	(21.8)	0.0239
Major Psychiatric Dis.	150	(6.5)	550	(6.0)	0.0181	134	(6.2)	228	(5.3)	0.0393
Myocardial Infarction	143	(6.2)	0	(0.0)	0.3628	0	(0.0)	0	(0.0)	
Obesity	228	(9.8)	467	(5.1)	0.1800	205	(9.4)	373	(8.6)	0.0292
Pre-Hospital Arrest	19	(0.8)	22	(0.2)	0.0797	18	(0.8)	22	(0.5)	0.0394
Premature	0	(0.0)	3	(0.0)	0.0257	0	(0.0)	0	(0.0)	
PVD	9	(0.4)	17	(0.2)	0.0377	6	(0.3)	11	(0.3)	0.0044
Respiratory Disease	159	(6.9)	464	(5.1)	0.0747	133	(6.1)	246	(5.7)	0.0191
Steroid Use	7	(0.3)	26	(0.3)	0.0031	5	(0.2)	9	(0.2)	0.0048

Data are presented as number (percentage) or mean [standard deviation].

SMD, standardized mean difference, ADD, Attention Deficit Disorder, CHF, Congestive Heart Failure, CVA, Cerebrovascular Accident, PVD, Peripheral Vascular Disease.

White patients were less likely to die than nonwhite patients (OR 0.7, 95%CI 0.6–0.9, p < 0.01) following splenectomy. Patients whose primary race was neither Black nor white were more likely to die (OR 1.3, 95%CI 1.03–1.7, p = 0.03). Patients who were uninsured (payment status listed as “self-pay”) were more likely to die (OR 1.6, 95%CI 1.3–1.9, p < 0.01), while patients with private insurance (OR 0.5, 95%CI 0.4–0.6, p < 0.01) or Medicaid (OR 0.6, 95%CI 0.5–0.8, p < 0.01) were less likely to die. Patients who were treated at an ACS-verified trauma center were also less likely to die (OR 0.4, 95%CI 0.3–0.7, p < 0.01). Geographical region was not associated with mortality.

Patients who were white were less likely to have three or more complications (OR 0.7, 95%CI 0.6–0.9, p < 0.01). Patients who were Black were more likely to have three or more complications (OR 1.7, 95%CI 1.3–2.0, p < 0.01) or a major complication (OR 1.3, 95%CI 1.1–1.4, p < 0.01). Patients with private insurance were less likely to have a major complication (OR 0.9, 95%CI 0.8–0.98, p = 0.02). Patients with Medicaid were more likely to have three or more complications (OR 1.4, 95%CI 1.2–1.8, p < 0.01). Patients who were treated in the southern region of the United States were more likely to have three or more complications (OR 1.5, 95%CI 1.2–1.7, p < 0.01) or a major complication (OR 1.4, 95%CI 1.3–1.6, p < 0.01) when compared with other geographic regions. Patients who were treated in the western region were less likely to have three or more complications (OR 0.7, 95%CI 0.5–0.9, p < 0.01) and less likely to have a major complication (OR 0.7, 95%CI 0.6–0.7, p < 0.01).

Among 5931 patients treated at hospitals with more than 600 beds, the post-splenectomy mortality rate was 10.4% (n = 615), 9.4% (n = 555) experienced three or more complications, and 22.8% (n = 1355) experienced a major complication. After propensity score matching, 549 patients who died prior to discharge were matched with 1084 controls, 533 patients who experienced three or more complications were matched with 1048 controls, and 1189 patients who experienced major complications were matched with 2368 controls. A summary of characteristics before and after matching can be found in Table 3, Table 4.

**Table 3:** Baseline characteristics before and after matching expired patients – hospitals with >600 beds following splenectomy.

Variables	Pre-Matching					Post-Matching				
	Expired (*n* = 549)		Controls (*n* = 5009)		SMD	Expired (*n* = 549)		Controls (*n* = 1084)		SMD
Age, y	49.06	[20.91]	40.82	[16.90]	0.3008	49.06	[20.91]	49.03	[18.59]	0.0013
Gender, male	377	(68.67)	3613	(72.13)	0.2769	377	(68.67)	734	(67.71)	0.0206
Injury Severity Score	36.54	[18.80]	30.97	[18.27]	0.4336	36.54	[18.80]	36.50	[18.00]	0.0023
Systolic Blood Pressure	104.45	[44.29]	114.99	[30.60]	0.0758	104.45	[44.29]	105.45	[32.30]	0.0257
Attention Deficit Disorder	30	(5.46)	491	(9.80)	0.1638	30	(5.46)	63	(5.81)	0.0150
Alcohol Use Disorder	0	(0.00)	17	(0.34)	0.0567	0	(0.00)	0	(0.00)	
Angina	0	(0.00)	9	(0.18)	0.1301	0	(0.00)	0	(0.00)	
Ascites	4	(0.73)	16	(0.32)	0.0203	4	(0.73)	8	(0.74)	0.0011
Bleeding Disorder	35	(6.38)	178	(3.55)	0.0632	35	(6.38)	67	(6.18)	0.0080
Chemotherapy	1	(0.18)	14	(0.28)	0.1180	1	(0.18)	2	(0.18)	0.0005
Congestive Heart Failure	17	(3.10)	68	(1.36)	0.4767	17	(3.10)	33	(3.04)	0.0030
Cirrhosis	22	(4.01)	65	(1.30)	0.0285	22	(4.01)	36	(3.32)	0.0365
Congenital Defect	0	(0.00)	10	(0.20)	0.1066	0	(0.00)	0	(0.00)	
Smoker	41	(7.47)	1225	(24.46)	0.1179	41	(7.47)	84	(7.75)	0.0106
CVA/Stroke	13	(2.37)	50	(1.00)	0.0788	13	(2.37)	23	(2.12)	0.0166
Dementia	5	(0.91)	18	(0.36)	0.0125	5	(0.91)	5	(0.46)	0.0544
Functionally Dependent	3	(0.55)	37	(0.74)	0.0240	3	(0.55)	1	(0.09)	0.0805
Diabetic	56	(10.20)	346	(6.91)	0.0600	56	(10.20)	120	(11.07)	0.0282
Dialysis	5	(0.91)	33	(0.66)	0.0645	5	(0.91)	13	(1.20)	0.0282
Disseminated Cancer	5	(0.91)	15	(0.30)	0.0150	5	(0.91)	9	(0.83)	0.0087
Drug Use	12	(2.19)	502	(10.02)	0.0419	12	(2.19)	29	(2.68)	0.0318
Esophageal Varices	1	(0.18)	12	(0.24)	0.0200	1	(0.18)	1	(0.09)	0.0243
Hypertension	109	(19.85)	912	(18.21)	0.0296	109	(19.85)	213	(19.65)	0.0051
Major Psychiatric Disorder	12	(2.19)	366	(7.31)	0.0201	12	(2.19)	28	(2.58)	0.0260
Myocardial Infarction	12	(2.19)	67	(1.34)	0.0341	12	(2.19)	20	(1.85)	0.0242
Obesity	31	(5.65)	318	(6.35)	0.1691	31	(5.65)	67	(6.18)	0.0226
Pre-Hospital Arrest	9	(1.64)	14	(0.28)	0.0694	9	(1.64)	10	(0.92)	0.0637
Premature	0	(0.00)	1	(0.02)	0.2425	0	(0.00)	0	(0.00)	
Peripheral Vascular Disease	2	(0.36)	14	(0.28)	0.3317	2	(0.36)	4	(0.37)	0.0008
Respiratory Disease	36	(6.56)	304	(6.07)	0.1397	36	(6.56)	64	(5.90)	0.0270
Steroid Use	1	(0.18)	18	(0.36)	0.0825	1	(0.18)	1	(0.09)	0.0243

Data are presented as number (percentage) or mean [standard deviation].

SMD, standardized mean difference, CVA, Cerebrovascular Accident.

**Table 4:** Baseline characteristics before and after matching patients with major complications following splenectomy – hospitals with >600 beds.

Variables	Pre-Matching					Post-Matching				
	Expired (*n* = 1268)		Controls (*n* = 4290)		SMD	Expired (*n* = 1189)		Controls (*n* = 2368)		SMD
Age, y	46.04	[18.54]	40.33	[16.98]	0.3214	44.91	[18.34]	44.27	[17.66]	0.0354
Gender, male	941	(74.21)	3049	(71.07)	0.0704	882	(74.18)	1739	(73.44)	0.0169
Injury Severity Score	35.69	[17.75]	30.29	[18.41]	0.2989	36.18	[17.51]	35.99	[17.29]	0.0110
Systolic Blood Pressure	110.41	[36.44]	115.00	[30.98]	0.1357	110.23	[36.72]	110.69	[32.27]	0.0134
ADD	147	(11.59)	374	(8.72)	0.0953	143	(12.03)	277	(11.70)	0.0102
Alcohol Use Disorder	11	(0.87)	9	(0.21)	0.0899	10	(0.84)	9	(0.38)	0.0592
Angina	85	(6.70)	128	(2.98)	0.1739	70	(5.89)	105	(4.43)	0.0657
Ascites	5	(0.39)	10	(0.23)	0.0288	4	(0.34)	7	(0.30)	0.0073
Bleeding Disorder	5	(0.39)	5	(0.12)	0.0550	4	(0.34)	5	(0.21)	0.0240
Chemotherapy	41	(3.23)	44	(1.03)	0.1533	35	(2.94)	41	(1.73)	0.0803
CHF	253	(19.95)	1013	(23.61)	0.0887	237	(19.93)	472	(19.93)	0.0000
Cirrhosis	10	(0.79)	28	(0.65)	0.0161	9	(0.76)	14	(0.59)	0.0202
Congenital Defect	27	(2.13)	36	(0.84)	0.1068	20	(1.68)	33	(1.39)	0.0234
Smoker	156	(12.30)	246	(5.73)	0.2308	139	(11.69)	220	(9.29)	0.0784
CVA/Stroke	7	(0.55)	13	(0.30)	0.0382	6	(0.50)	7	(0.30)	0.0331
Dementia	5	(0.39)	8	(0.19)	0.0386	5	(0.42)	8	(0.34)	0.0134
Functionally Dependent	12	(0.95)	28	(0.65)	0.0330	11	(0.93)	21	(0.89)	0.0040
Diabetic	4	(0.32)	5	(0.12)	0.0428	2	(0.17)	4	(0.17)	0.0002
Dialysis	79	(6.23)	0	(0.00)	0.3644	0	(0.00)	0	(0.00)	
Disseminated Cancer	7	(0.55)	9	(0.21)	0.0556	4	(0.34)	8	(0.34)	0.0002
Drug Use	312	(24.61)	709	(16.53)	0.2008	264	(22.20)	497	(20.99)	0.0295
Esophageal Varices	0	(0.00)	1	(0.02)	0.0216	0	(0.00)	0	(0.00)	
Hypertension	123	(9.70)	226	(5.27)	0.1690	112	(9.42)	187	(7.90)	0.0541
Major Psychiatric Dis.	99	(7.81)	241	(5.62)	0.0876	81	(6.81)	144	(6.08)	0.0298
Myocardial Infarction	5	(0.39)	14	(0.33)	0.0113	4	(0.34)	10	(0.42)	0.0140
Obesity	33	(2.60)	54	(1.26)	0.0977	30	(2.52)	48	(2.03)	0.0333
Pre-Hospital Arrest	8	(0.63)	15	(0.35)	0.0403	7	(0.59)	11	(0.46)	0.0172
Premature	95	(7.49)	283	(6.60)	0.0350	86	(7.23)	169	(7.14)	0.0037
PVD	91	(7.18)	423	(9.86)	0.0962	88	(7.40)	167	(7.05)	0.0135
Respiratory Disease	10	(0.79)	13	(0.30)	0.0659	10	(0.84)	13	(0.55)	0.0351
Steroid Use	3	(0.24)	14	(0.33)	0.0169	3	(0.25)	8	(0.34)	0.0158

Data are presented as number (percentage) or mean [standard deviation].

SMD, standardized mean difference, ADD, Attention Deficit Disorder, CHF, Congestive Heart Failure, CVA, Cerebrovascular Accident, PVD, Peripheral Vascular Disease.

**Fig. 1:**
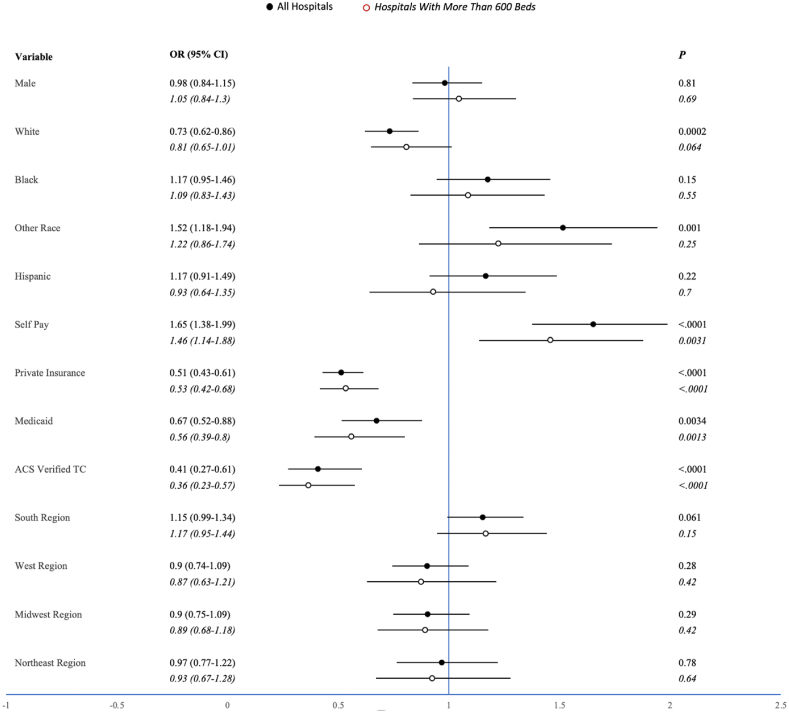
Mortality Risk Ratios – Odds ratios with 95% confidence intervals and p-values visualized both before and after limiting analysis to hospitals with greater than 600 beds for each variable’s effect on mortality.

**Fig. 2:**
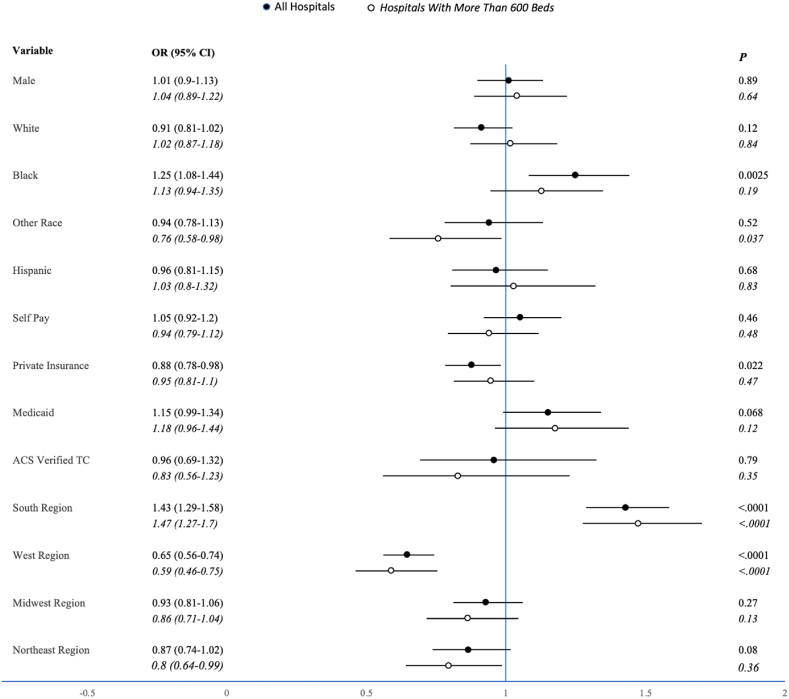
Major Complication Risk Ratios – Odds ratios with 95% confidence intervals and p-values visualized both before and after limiting analysis to hospitals with greater than 600 beds for each variable’s effect on major complications.

There were no significant associations between race and mortality in patients treated at hospitals with over 600 beds. Patients with private insurance (OR 0.5, 95%CI 0.4–0.7, p < 0.0001) or Medicaid (OR 0.6, 95%CI 0.4–0.8, p = 0.0013), or those treated at an ACS-verified trauma center (OR 0.4, 95%CI 0.2–0.6, p < 0.0001) were less likely to die. Patients without insurance (OR 1.5, 95%CI 1.1–1.9, p = 0.004) were more likely to die. Patients with a primary race of Black were more likely to experience three or more complications (OR 1.58, 95%CI 1.21–2.07, p = 0.0007). Patients with a primary race other than Black or white were less likely to experience a major complication (OR 0.76, 95%CI 0.6–0.98, p = 0.037). Neither private insurance nor self-pay were correlated with complications. Patients with Medicaid were more likely to experience three or more complications (OR 1.4, 95%CI 1.02–1.8, p = 0.037). Patients treated in the South were more likely to experience three or more complications (OR 1.5, 95%CI 1.2–1.8, p = 0.0008) or a major complication (OR 1.5, 95%CI 1.3–1.7, p < 0.0001). Patients treated in the Midwest were less likely to experience three or more complications (OR 0.7, 95%CI 0.6–0.99, p = 0.043), and those treated in the West were less likely to experience a major complication (OR 0.6, 95%CI 0.5–0.8, p < 0.0001). A summary of these results may be found in Fig. 1, Fig. 2.

## 4 Discussion

The present study demonstrates that disparities in outcomes exist between patients of different races, payment status, and geographical regions who underwent a splenectomy at a trauma center. Hospital volume has been shown to correlate with higher quality outcomes [11]. Accordingly, limiting our analysis to hospitals with over 600 beds led to reduced racial- and insurance-related disparities in outcomes. Again, race itself is not a risk factor but is associated with many other social, economic, and clinical factors that affect medical outcomes [12].

### 4.1 Race

Mortality and other complications were less frequent in patients who were white. A primary race of Black was not associated with mortality frequency, but it was associated with a higher frequency of multiple complications and major complications. Mortality was also more frequent among patients who were neither white nor Black. Among patients treated at high-volume hospitals, there were no significant associations between race and mortality. A primary race of Black was associated only with multiple complications, and a primary race other than Black or white was associated only with a higher risk of a major complication. This supports similar findings by Breslin et al. [18], who showed that mortality rates were not associated with race within hospitals with large minority populations, and that hospital factors account for racial disparities in mortality rates in breast and colon cancer patients. Multiple other studies have shown that controlling for the quality of individual hospitals can help to remove disparities in outcome, and that there are often associations between demographics and the hospital at which a patient is treated [19,20]. Our findings suggest that hospital factors can account for racial disparities in trauma patients who require a splenectomy as well, and further research could investigate whether this can be generalized to all trauma patients.

### 4.2 Payment status

Mortality was less frequent in patients with private insurance and more frequent in patients without insurance. Medicaid patients had a lower frequency of mortality but a higher frequency of multiple complications. Of note, payment information was unavailable for 2622 of the 11,419 patients (23.0%), including patients who were not billed for any reason, received workers' compensation, or were involved in no-fault automobile accidents. Haines et al. [21] produced comparable findings showing that uninsured patients were more likely to die, while Medicaid was not associated with mortality. Haines et al. [21] did not consider complications as an end point but did find that Medicaid was associated with a longer hospital stay.

We found that associations between payment status and outcome were diminished when limited to high-volume hospitals but not eliminated. Self-pay was still significantly associated mortality risk, but the association was stronger when all hospitals were included. Self-pay and private insurance were no longer associated with multiple complications or major complications, but Medicaid still had a significant association with multiple complications.

### 4.3 Geographical regions

While the geographical location of a trauma center was not associated with mortality, we did find geographical associations with multiple or major complications in our dataset. Multiple complications and major complications were more frequent in patients treated at trauma centers in the South of the U.S., while multiple complications were less frequent in patients treated in the West. Major complications were less frequent in patients treated in the Midwest. Limiting our analysis to larger hospitals had a relatively small effect on geographical associations with outcome and in some cases resulted in associations that were not present beforehand. Brown et al. [22] showed that the geographic distribution of trauma centers is correlated with mortality, and states with dispersed trauma centers (many of which are in the South) have longer transport times and higher mortality rates than states with clustered trauma centers. Lastly, we did find that mortality was less frequent in patients treated at an ACS-verified trauma center. Of note, only 3.4% (n = 105) of the patients in this propensity-score-matched cohort were treated at non-ACS-verified trauma centers.

### 4.4 Limitations

There are multiple limitations to our analysis that must be noted. Comorbidity data in the TQIP dataset is only available in a binary form, and it is not possible to account for differences in the severity of preexisting comorbidities. For example, we found that obesity and hypertension were associated with outcomes, but we were only able to match patients based on either the presence or absence of these comorbidities and not the severity. It has also been shown that there are geographical differences in the presence of comorbidities, such as obesity, throughout the United States [23].

Previous studies have shown that many of these demographics, especially race and payment status, are related and cannot be analyzed as completely independent variables [24]. It has also been shown that inclusion of nonsurvivable injuries in the TQIP database might account for differences in outcomes between trauma centers, which could be one factor in the differences in outcomes between the different regions, though our propensity-matching process did account for injury severity score [25]. Additionally, hospital bed count is not a direct measurement of the volume of trauma patients treated, and there are some very high-volume trauma centers in hospitals with fewer than 600 beds.

### 4.5 Social determinants of health

As early as 1977, when Bronfenbrenner et al. [26] proposed a research model that considers the changing environment in which a human develops, it has been recognized that there are a multitude of factors affecting the health of an individual. The National Institute on Minority Health and Health Disparities Research Framework proposed by Alvidrez et al. [27] serves as a model for ensuring that research addresses the complexity of health disparities and the determinants that affect them. The framework proposes four levels of influence: individual, interpersonal, community, and societal. The data available in the TQIP database allows us to examine only some of the factors on the individual level of influence, namely race and insurance status. We were not able to examine the other three levels beyond individual, which include factors ranging from family functioning, patient-clinician relationship, and availability of health services in a community to sanitization, immunization, societal norms, and quality of care. Additionally, insurance status is an incomplete marker of socioeconomic status, and this is a significant limitation. There is a need for continued analysis with a more specific data collection that can provide a more complete picture of the social determinants of health.

Even though we are limited by the available data points, our findings do suggest that racial- and insurance-related disparities in outcomes can be reduced by controlling for certain hospital characteristics, such as, in this case, size. Providing necessary quality improvements to hospitals that treat a larger number of minority patients, which has previously been proposed by Osborne et al. [28], is likely to reduce the disparities in outcomes. This is not a complete solution, and ultimately many other social determinants of health must be addressed to eliminate disparities in outcomes. It is also important to note that the term “lower-quality hospital” does not imply fault among those who staff those facilities but indicates a multitude of factors including a lack of resources and less access to specialized care. Bach et al. [29] found that physicians primarily treating Black patients had significantly less access to high-quality imaging and subspecialists, and that those physicians were less likely to be board certified.

## 5 Conclusion

After controlling for preexisting medical conditions and injury severity in a cohort of 11,419 patients who underwent a splenectomy in a U.S. trauma center from 2010 to 2015, race and payment status independently predicted mortality and in-hospital complications. A primary race of Black predicted multiple complications and major complications, while a primary race of white was associated with lower risks of mortality and a lesser percentage with multiple complications. A lack of health insurance predicted mortality while private insurance was associated with a lower risk of both mortality and major complications. These disparities are reduced among high-volume hospitals. A major implication of this study is that improving the quality of lower-volume hospitals and those that treat a larger proportion of minority patients can reduce disparities in outcomes.

## Authorship

HJK and IML conceived of the study concept and design. HJK contributed to data acquisition and analysis. HJK wrote the original manuscript draft and IML provided critical revision.

## Please state any sources of funding for your research

None to declare.

## Ethical approval

This study was reviewed by the Institutional Review Board of the Icahn School of Medicine at Mount Sinai in New York, NY (IRB-20-03069) including a waiver of patient consent.

## Consent

N/A.

## Author contribution

Study Design and Concept – Harrison J. Kaplan and Dr. I. Michael Leitman.

Data Collection – Dr. I. Michael Leitman.

Data Analysis and Interpretation – Harrison J. Kaplan.

Writing of manuscript – Harrison J. Kaplan and Dr. I Michael Leitman.

## Registration of research studies

1. Name of the registry: Research Registry.

2. Unique Identifying number or registration ID: researchregistry7562.

3. Hyperlink to your specific registration (must be publicly accessible and will be checked): http://www.researchregistry.com.

## Guarantor

Harrison J. Kaplan and I. Michael Leitman.

## Declaration of competing interest

This research did not receive any specific grant from funding agencies in the public, commercial, or not-for-profit sectors.
